# IGF2BP3-mediated enhanced stability of MYLK represses MSC adipogenesis and alleviates obesity and insulin resistance in HFD mice

**DOI:** 10.1007/s00018-023-05076-0

**Published:** 2024-01-10

**Authors:** Xiuji Huang, Wuhui He, Shuai Fan, Hui Li, Guiwen Ye

**Affiliations:** 1 Department of Respiratory and Critical Care Medicine, The Seventh Affiliated Hospital, Sun Yat-sen University, Shenzhen, 518107 People’s Republic of China; 2 Department of Orthopedics, The Eighth Affiliated Hospital, Sun Yat-sen University, Shenzhen, 518033 People’s Republic of China; 3 Department of Otolaryngology, Sun Yat-sen Memorial Hospital, Sun Yat-sen University, Guangzhou, 510120 People’s Republic of China

**Keywords:** Mesenchymal stem cells, Adipogenesis, m6A modification

## Abstract

Mesenchymal stem cells (MSCs) hold immense potential as multipotent stem cells and serve as a primary source of adipocytes. The process of MSC adipogenesis plays a crucial role in maintaining systemic metabolic homeostasis and has garnered significant attention in tissue bioengineering. N6-methyladenosine (m6A), the most prevalent RNA modification, is known to regulate cell fate and disease. However, the precise involvement of m6A readers in MSC adipogenesis remains unclear. In this study, we investigated the impact of IGF2BP3, a prominent m6A reader, on MSC adipogenesis. Our findings revealed a decrease in IGF2BP3 expression during the natural adipogenic differentiation of MSCs. Furthermore, IGF2BP3 was found to repress MSC adipogenesis by augmenting the levels of MYLK, a calcium/calmodulin-dependent kinase. Mechanistically, IGF2BP3 interacted with MYLK mRNA in an m6A-dependent manner, extending its half-life and subsequently inhibiting the phosphorylation of the ERK1/2 pathway, thereby impeding the adipogenic differentiation of MSCs. Additionally, we successfully achieved the overexpression of IGF2BP3 through intraperitoneal injection of adeno-associated virus serotype Rec2, which specifically targeted adipose tissue. This intervention resulted in reduced body weight and improved insulin resistance in high-fat diet mice. Overall, our study provides novel insights into the role of IGF2BP3 in MSC adipogenesis, shedding light on adipocyte-related disorders and presenting potential targets for related biomedical applications.

**Supplementary Information:**

The online version contains supplementary material available at 10.1007/s00018-023-05076-0.

## Introduction

Mesenchymal stem cells (MSCs) are a kind of stem cell with multidirectional differentiation potential, including adipogenesis, osteogenesis and chondrogenesis [1]. In recent years, MSCs have garnered significant attention from scientists due to their involvement in numerous pathophysiological processes and their potential applications in tissue regeneration engineering [2]. The regulation of differentiation fate is a critical aspect of MSC function in mediating disease occurrence and clinical applications [3]. MSC adipogenesis, in particular, plays a pivotal role as the primary source of adipocytes, which are crucial for systemic metabolic homeostasis and implicated in various diseases such as type 2 diabetes, cardiovascular diseases, and osteoporosis [4, 5]. Furthermore, MSC adipogenesis has found applications in tissue bioengineering, particularly in soft tissue padding and aesthetic medicine [6, 7]. Previously, some studies have explored the mechanism and application of MSC adipogenesis [8]. However, studies are still required to further clarify the regulatory network of MSC adipogenesis.

N6-methyladenosine (m6A) is the most abundant type of RNA modification that participates in the regulation of cell fate and disease development [9]. The m6A modification is dynamically regulated by transmethylases such as METTL3 and demethylases such as FTO [9]. Importantly, m6A readers, such as YTHDF2, IGF2BP1 and IGF2BP3, recognize the m6A modification on RNA and modulate their characteristics [10]. IGF2BP3, insulin-like growth Factor 2 mRNA binding protein 3, is capable of enhancing the stability or translation efficiency of mRNA [11]. Previous studies have reported that m6A readers participate in adipogenesis and adipocyte-related disorders. YTHDF2 captures the higher m6A level of Atg5 transcripts mediated by FTO silencing and inhibited adipogenesis [12]. IGF2BP1 is induced in abdominal fat by a high-fat diet (HFD) and can promote adipocyte differentiation [13]. However, few studies have comprehensively investigated the role of m6A readers in the natural adipogenic differentiation of MSCs.

MYLK, myosin light chain kinase, is a calcium/calmodulin-dependent kinase that mainly participates in protein phosphorylation and myosin interaction [14]. Previous studies have reported that MYLK is involved in the regulation of cellular differentiation and stem migration. Leitman and his colleagues revealed that MYLK regulates Schwann cell cytoskeletal organization and differentiation [15]. Lin and colleagues showed that MYLK engaged in inflammatory cytokine-mediated MSC migration [16]. However, the function of MYLK in MSC differentiation has not been evaluated.

In our study, we found that the expression of IGF2BP3 decreased gradually during adipogenic induction and that IGF2BP3 repressed MSC adipogenesis by binding MYLK mRNA in a m6A manner and strengthening the stability of MYLK, which blocked the activation of the ERK1/2 pathway. In addition, specifically overexpressing IGF2BP3 in adipocytes by adeno-associated virus serotype Rec2 (AAVRec2) decreased body weight and alleviated insulin resistance in HFD mice. Our findings revealed valuable insights into MSC adipogenesis and provided potential targets for related applications.

## Materials and methods

### MSC isolation and culture

The MSCs used in this study were isolated and cultured according to previously described methods [17]. Fresh human bone marrow was extracted from the posterior superior iliac spine of twelve healthy donors. The isolated MSCs were cultured in Dulbecco's Modified Eagle's Medium (DMEM; Gibco) containing 10% fetal bovine serum (FBS; Gibco) at 37 °C with 5% CO_2_, and the culture medium was replaced every 3 days. MSCs were passaged when they reached approximately 90% confluence and were used in experiments at passage 4.

### Induction of adipogenic differentiation

Adipogenic induction medium was prepared by supplementing DMEM with 10% FBS, 0.5 mM IBMX (Sigma), 1 mM dexamethasone (Sigma), 10 mg/ml insulin (Sigma), 0.2 mM indomethacin (Sigma), 100 IU/ml penicillin (Sigma), and 100 IU/ml streptomycin (Sigma). MSCs were seeded on culture plates and cultured with adipogenic induction medium, with medium replacement every 3 days until detection.

### Oil red O (ORO) staining

ORO staining was performed on day 14 after adipogenic induction. Cells were fixed with 4% paraformaldehyde for 20 min and stained with fresh ORO solution for 15 min. The ORO solution was then discarded, and the cells were gently washed with phosphate-buffered saline (PBS) three times. Finally, the cells were observed and captured using a microscope. Subsequently, the cells were destained with isopropyl alcohol, and the absorbance of the extracted liquor was measured using a Thermo Scientific Microplate Reader at 520 nm.

### real-time quantitative polymerase chain reaction (RT-qPCR)

Total RNA from MSCs was extracted using TRIzol™ Reagent (Thermo Fisher, 15596-026) and reverse transcribed into cDNA using Evo M-MLV RT Master Mix [ACCURATE BIOTECHNOLOGY (HUMAN), AG11706, Changsha, China] according to the manufacturer's instructions. The cDNA was then used for RT-qPCR using SYBR® Green Pro Taq HS Premix (Accurate Biology, AG11701) on a real-time system (Bio-Rad, CFX96 Touch). The relative expression levels were analyzed using the 2^−ΔΔCt^method with GAPDH as the reference gene. The primers used in this study are listed in Table S1.

### Western blot

Proteins from MSCs were lysed using RIPA buffer and collected by centrifugation at 14,000 rpm for 10 min at 4 °C. The protein lysates' concentrations were determined using the BCA Protein Assay Kit (CWBIO, CW0014S), and equal amounts of protein lysate were mixed with sodium dodecyl sulfate loading buffer. The mixtures were boiled for 10 min and used for western blot detection. Omni-PAGE™ Hepes-Tris Gels (EpiZyme, LK214) were prepared, and the protein lysates were added and separated via electrophoresis. The proteins on the gels were then transferred to polyvinylidene fluoride membranes (Millipore, IPVH0010). The membranes were blocked with 5% non-fat milk and incubated with primary antibodies overnight at 4 °C. Subsequently, the membranes were incubated with secondary antibodies for 1 h at room temperature, and the protein signals were detected using Immobilon Western HRP Substrate (Millipore, WBKLS0500). The images were analyzed using ImageJ, and the relative expression was calculated based on the gray values, with GAPDH as the endogenous reference gene. The antibodies used in this study are listed in Table S2.

### RNA interference and lentivirus transfection

Small interfering RNAs (siRNAs) and lentiviruses were designed and constructed by IGE Biotechnology (Guangzhou, China). The siRNAs were transfected using Lipofectamine RNAiMAX (Thermo), and the lentiviruses were transfected using polybrene according to the manufacturer's instructions. The "control" group represents cells treated with transfection reagents without siRNA or lentivirus. The interfering efficiency of siRNA was assessed by RT-qPCR 48 h later and by western blot 72 h later. The transfection efficiency of lentivirus was evaluated by RT-qPCR 72 h later and by western blot 96 h later. The sequences of the siRNAs used in this study are listed in Table S3.

### In vivo adipogenesis assay

The experiment was conducted following previously described methods [18]. MSCs were subjected to interference using siRNAs or lentivirus-mediated infection and cultured in adipogenic induction medium for 5 days in vitro. On day 6, cells from each group were collected and suspended in Matrigel (BD Biosciences, USA) at a concentration of 1.5 × 10^5^ cells/mL. For the implantation procedure, 8-week-old male nude mice were anesthetized using 10 mL/kg of 4% chloral hydrate. Subcutaneous grafting was performed by injecting 150 μL of Matrigel containing si-IGF2BP3 MSCs or ov-IGF2BP3 MSCs on one side of the mice's back, while 150 μL of Matrigel containing negative control cells was grafted symmetrically on the other side of the mice's back (*n* = 4 per group). After 8 weeks, the grafts were collected, fixed in 10% neutral formalin for 24 h, embedded in paraffin, and sliced for H&E staining or immunohistochemistry (IHC).

### HE staining and immunohistochemistry

Firstly, the tissue sections were deparaffinized using xylene and then hydrated with ethanol prior to staining. For H&E staining, the sections were stained with hematoxylin for 5 min, followed by rinsing with running water and subsequent staining with eosin for 3 min. The stained sections were then sealed for observation under an optical microscope.

For Immunohistochemistry (IHC) staining, an SP Rabbit & Mouse HRP Kit (CWBIO, CW2069) was used according to the manufacturer's instructions. The sections underwent antigen retrieval using pepsase for 20 min. Subsequently, the sections were blocked with 10% goat serum for 30 min and incubated overnight at 4 °C with anti-Perilipin-1 antibody (Abcam, ab3526, 1:100). On the following day, the sections were incubated with secondary antibodies and a DAB substrate. Finally, the sections were counterstained with hematoxylin and sealed for observation under an optical microscope.

### RNA sequencing

MSCs from four different donors were interfered with either si-NC or si-IGF2BP3, and two days later, the total RNA was extracted from each group using Trizol solution. After quality control, the RNA was fragmented and reverse-transcribed into cDNA. The RNA library was constructed using the NEBNext® Ultra™ RNA Library Prep Kit for Illumina (New England Biolabs, NEB #E7770). Finally, sequencing was performed using the Illumina HiSeq at Qiantang Biotechnology (Suzhou) Co., Ltd.

### RNA immunoprecipitation (RIP)

RIP was performed using the EZ-Magna RIP™ RNA-Binding Protein Immunoprecipitation Kit (Millipore, 17–701) following the manufacturer's instructions. MSCs were lysed with RIPA buffer and incubated with magnetic beads conjugated with specific antibodies, including m6A methylation antibody (Synaptic Systems, 202003, 1:100), anti-IGF2BP3 (Abcam, ab177477, 1:100), anti-METTL3 (Abcam, ab195352, 1:50), anti-FTO (Abcam, ab177477, 1:100), or negative control IgG (Santa Cruz, sc-3877, 1:100). The immunoprecipitated RNA was then extracted and reverse-transcribed. Finally, the abundance of MYLK mRNA was measured by RT-qPCR.

### RNA pulldown

Plasmids overexpressing MYLK mRNA or relevant antisense transcripts were constructed by IGE Biotechnology (Guangzhou, China). The MYLK mRNA or antisense transcripts were transcribed using the TranscriptAid T7 High-Yield Transcription Kit (Thermo Fisher, K0441). RNA pulldown assays were performed using the Pierce™ Magnetic RNA-Protein Pull-Down Kit (Thermo Fisher, 20164) following the manufacturer's instructions. The proteins binding to RNA were purified, and their abundances were detected by western blotting.

### RNA stability detection

MSCs were seeded on culture plates and treated with siRNAs or lentiviruses. After three days, 2 µg/ml of actinomycin D was added to inhibit transcription. The cells were then lysed with Trizol solution at various time points (0 min, 30 min, 60 min, 90 min, 120 min, 150 min, and 180 min). Total RNA was extracted, and cDNA was synthesized in reverse. The relative abundance of MYLK was measured by RT-qPCR, and the half-life period was calculated using GraphPad Prism.

### HFD mouse model construction and detection

Twelve male C57BL/6J mice were randomly divided into three groups: the normal control diet (NCD) group, the HFD + control AAV group (HFD-NC), and the HFD + IGF2BP3 AAV group (HFD-OV). HFD mouse models were fed a 60% high-fat forage (protein: 20%; fat: 60%; carbohydrate: 20%), while the NCD group was fed normal control forage. AAVRec2, constructed by OBiO Technology (Shanghai, China), was intraperitoneally injected at the beginning of model induction. At 8 weeks, the body weight, ITT, and HOMA-IR were tested, followed by GTT one week later. Afterward, the mice were sacrificed, and abdominal adipose tissues were collected. The abdominal adipose tissues were weighed to calculate the body fat ratio, and frozen slices were made for immunofluorescence detection.

### Statistical analyses

The data of this study were analyzed by SPSS 22.0 and presented as mean ± standard deviation (SD). The statistical analyses of differences between different groups were conducted using independent-sample *t*-tests or one-way ANOVA. *P* < 0.05 was regarded as significant. The sample sizes and the *p*-values were included in the figure legends.

## Results

### The level of IGF2BP3 was decreased during MSC adipogenesis

Firstly, we assessed the characteristics of the isolated MSCs. The results demonstrated that the cells exhibited positive expression for CD29, CD44, and CD105, while they were negative for CD14, CD45, and HLA-DR (Figure S1A). Furthermore, Alizarin Red S, ORO, and toluidine blue staining confirmed the osteogenic, adipogenic, and chondrogenic differentiation potential of the cells (Figure S1B). These findings indicated that the MSCs used in our study met the international criteria [19].

**Fig. 1:**
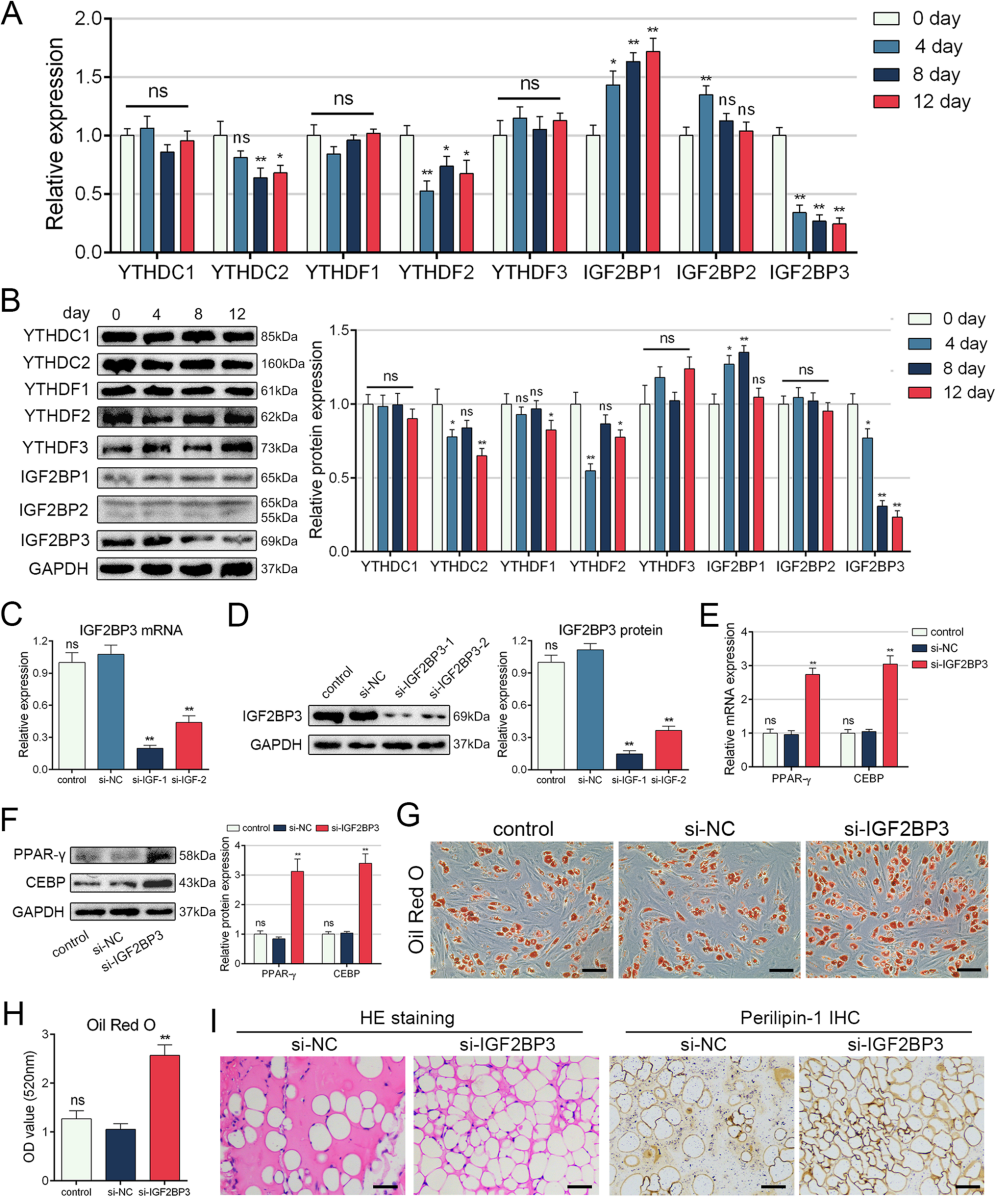
The level of IGF2BP3 decreased during MSC adipogenesis and knockdown of IGF2BP3 facilitated MSC adipogenesis. **A** The mRNA levels of m6A readers on day 0, day 4, day 8 and day 12 after adipogenic induction. **B** The protein levels of m6A readers on day 0, day 4, day 8 and day 12 after adipogenic induction. **C** Interference efficiency of IGF2BP3 siRNAs on mRNA level. **D** Interference efficiency of IGF2BP3 siRNAs on protein level. **E** Silencing IGF2BP3 enhanced mRNA levels of PPAR-γ and CEBP. **F** Silencing IGF2BP3 enhanced protein levels of PPAR-γ and CEBP. **G**, **H** Silencing IGF2BP3 enhanced the intensive of ORO staining. **I** Histological detection revealed increased fat vacuole formation and stronger intensity of perilipin-1 with IGF2BP3 knockdown. *n* = 12 (**A**–**H**), *n* = 6 (**I**), ns indicates not significant, * indicates *P* < 0.05, ** indicates *P* < 0.01, scale bar = 100 nm (**G**), scale bar = 50 nm (**I**)

To investigate the involvement of m6A readers in MSC adipogenesis, we examined the expression levels of key readers during different stages of MSC adipogenesis. The results revealed a reduction in the levels of YTHDC2, YTHDF2, and IGF2BP3, while the levels of IGF2BP1 and IGF2BP2 increased (Fig. 1A and B). Among them, IGF2BP3 exhibited a gradual and most pronounced decrease during adipogenic induction. Additionally, correlation analysis demonstrated that the expression levels of IGF2BP3 had the strongest correlation with the intensity of ORO staining (Figure S2). Therefore, we selected IGF2BP3 for further investigation into MSC adipogenesis.

### Knockdown of IGF2BP3 facilitated MSC adipogenesis

To investigate the role of IGF2BP3 in MSCs, we employed siRNAs to silence its expression, as depicted in Fig. 1C and D, demonstrating the efficiency of interference. The most effective siRNA was selected for further experiments. Subsequently, we examined the mRNA and protein levels of PPAR-γ and CEBP, two crucial adipogenic markers, and observed their increase upon IGF2BP3 knockdown (Fig. 1E and F). Moreover, ORO staining revealed enhanced adipocyte formation following IGF2BP3 silencing (Fig. 1G and H). To evaluate the impact of IGF2BP3 knockdown on MSC adipogenesis in vivo, we conducted adipogenic differentiation experiments on the backs of nude mice. The results demonstrated that IGF2BP3 knockdown in MSCs resulted in increased formation of fat vacuoles and stronger intensity of perilipin-1, a marker for fat droplets (Fig. 1I). Collectively, these findings indicate that knockdown of IGF2BP3 promotes MSC adipogenesis.

### Overexpression of IGF2BP3 repressed MSC adipogenesis

**Fig. 2:**
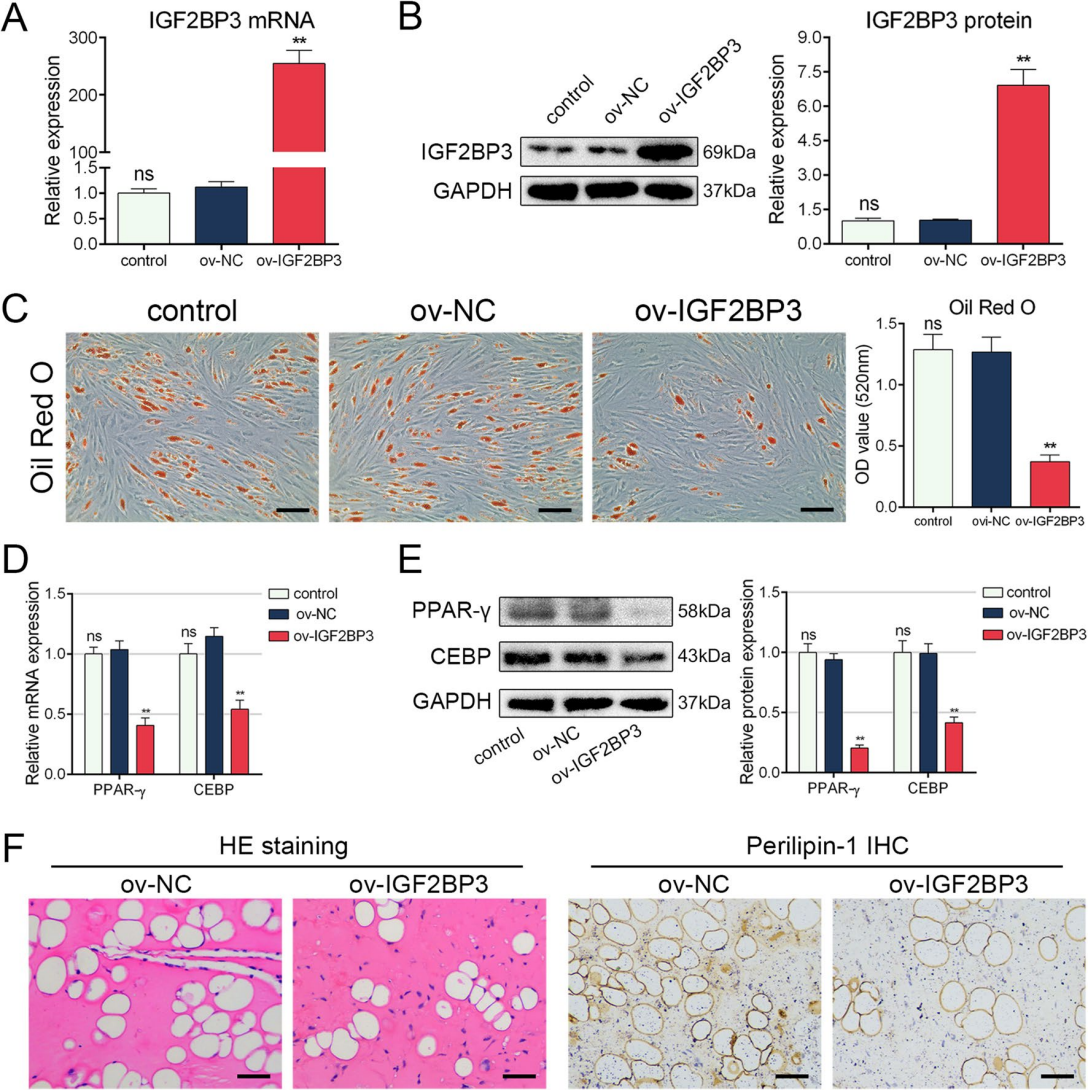
Overexpression of IGF2BP3 repressed MSC adipogenesis. **A** The overexpression efficiency of IGF2BP3 lentivirus on mRNA level. **B** The overexpression efficiency of IGF2BP3 lentivirus on protein level. **C** IGF2BP3 overexpression inhibited the intensity of ORO staining. **D** IGF2BP3 overexpression decreased the mRNA levels of PPAR-γ and CEBP. **E** IGF2BP3 overexpression decreased the protein levels of PPAR-γ and CEBP. **F** Histological detection showed less fat vacuole formation and weaker intensive of perilipin-1 with IGF2BP3 overexpression. *n* = 12 (**A** to **E**), *n* = 6 (**F**), ns indicates not significant, * indicates *P* < 0.05, ** indicates *P* < 0.01, scale bar = 100 nm (**C**), scale bar = 50 nm (**F**)

To further elucidate the impact of IGF2BP3 on MSC adipogenesis, we generated a lentivirus for IGF2BP3 overexpression, as illustrated in Fig. 2A and B, demonstrating the efficiency of overexpression. ORO staining revealed that the overexpression of IGF2BP3 inhibited the formation of adipocytes (Fig. 2C). Moreover, the mRNA and protein levels of adipogenic markers were also reduced upon IGF2BP3 overexpression (Fig. 2D and E). Additionally, an in vivo experiment conducted on nude mice demonstrated that IGF2BP3 overexpression in MSCs resulted in a decrease in fat vacuole formation and a weaker intensity of perilipin-1 (Fig. 2F). These findings clearly indicate that IGF2BP3 overexpression suppresses MSC adipogenesis.

### RNA-seq screened the downstream target gene of IGF2BP3

**Fig. 3:**
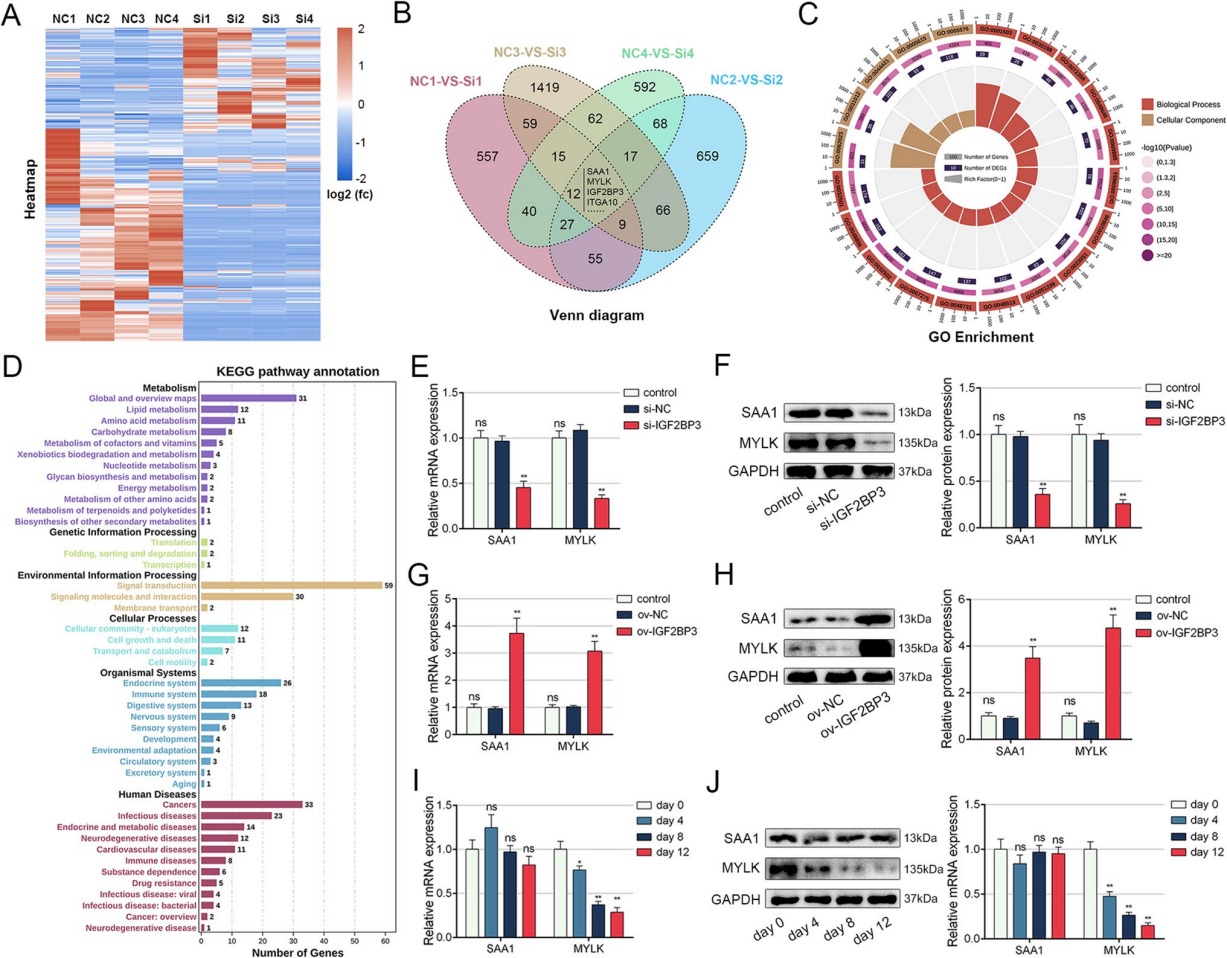
Identification of downstream genes of IGF2BP3 through RNA-seq analysis. **A** Heatmap representing the differentially expressed genes analyzed using the matched-pair analysis method. **B** Venn diagram showing the intersection of four sets of differentially expressed genes. **C** Top 20 enriched terms from the GO analysis. **D.** KEGG pathway annotation of the RNA-seq results. **E–F** IGF2BP3 knockdown reduced both the mRNA and protein levels of SAA1 and MYLK. **G–H** IGF2BP3 overexpression elevated both the mRNA and protein levels of SAA1 and MYLK. **I–J** The level of SAA1 remains unchanged, while the level of MYLK gradually decreases during MSC adipogenesis. *n* = 12 (**E** to **J**), ns indicates not significant, * indicates *P* < 0.05, ** indicates *P* < 0.01

To investigate the mechanism by which IGF2BP3 regulates MSC adipogenesis, we performed RNA-seq analysis on four MSCs treated with si-NC or si-IGF2BP3. The expression distribution of each sample is presented in Figure S3A-B. Matched-pair analysis revealed that IGF2BP3 knockdown resulted in the upregulation of 157 genes and the downregulation of 253 genes (Fig. 3A). The Venn diagram displayed an intersection of these four sets of differentially expressed genes, which included 12 genes, among them SAA1 and MYLK (Fig. 3B). GO analysis revealed the top 20 enriched biological process and cellular component terms (Fig. 3C). KEGG pathway analysis demonstrated that the differentially expressed genes were primarily associated with lipid metabolism, signal transduction, and the endocrine system (Fig. 3D). Subsequently, we screened for the downstream target gene of IGF2BP3 among the 12 genes in the Venn diagram. We selected the top 5 differentially expressed genes with higher FPKM values and verified the effect of IGF2BP3 on their expression. The results showed that IGF2BP3 knockdown significantly impaired the levels of SAA1 and MYLK (Fig. 3E and F), while IGF2BP3 overexpression had the opposite effect (Fig. 3G and H). The expression of CES1, CDKL5, and ITGA10 was not influenced by IGF2BP3 (Figure S4). Additionally, the level of SAA1 remained unchanged, whereas the level of MYLK gradually decreased during adipogenic induction (Fig. 3I and J). Collectively, these findings suggest that MYLK may be a downstream target gene of IGF2BP3 in the regulation of MSC adipogenesis.

### IGF2BP3 repressed MSC adipogenesis through MYLK

**Fig. 4:**
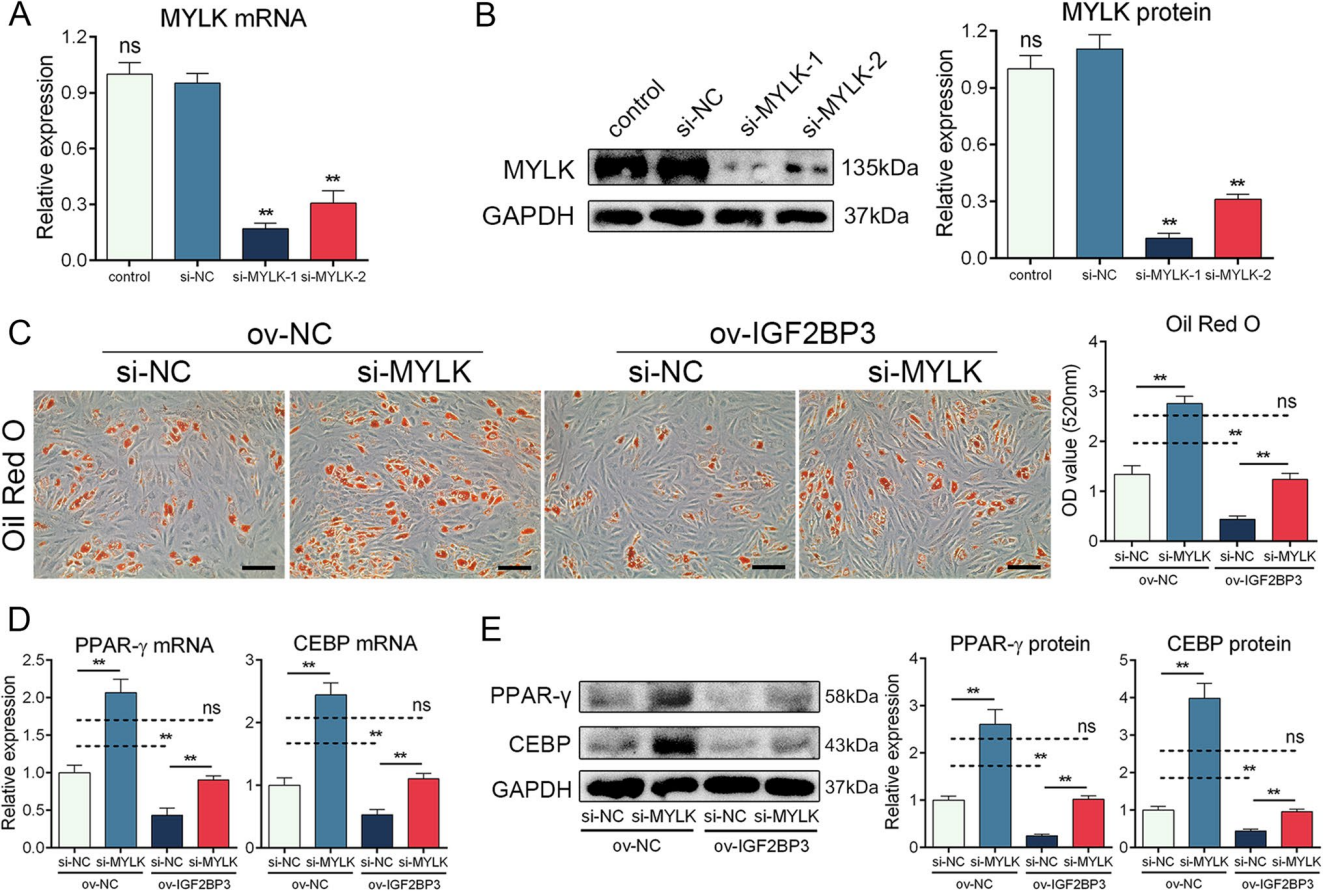
IGF2BP3 repressed MSC adipogenesis through MYLK. **A** Interference efficiency of MYLK siRNAs on mRNA level. **B** Interference efficiency of MYLK siRNAs on protein level. **C** Silencing MYLK promoted the intensity of ORO staining and reversed the effect of IGF2BP3 overexpression. **D** Silencing MYLK enhanced the mRNA levels of PPAR-γ and CEBP and reversed the effect of IGF2BP3 overexpression. **E** Silencing MYLK enhanced the proteins level of PPAR-γ and CEBP and reversed the effect of IGF2BP3 overexpression. *n* = 12, ns indicates not significant, * indicates *P* < 0.05, ** indicates *P* < 0.01, scale bar = 100 nm

To further investigate the role of MYLK in MSC adipogenesis, we employed siRNAs to silence MYLK in MSCs. The interference efficiency is presented in Fig. 4A and B, and the more effective siRNA was selected for subsequent experiments. ORO staining revealed that knockdown of MYLK enhanced the formation of adipocytes and reversed the effect of IGF2BP3 overexpression (Fig. 4C). Additionally, the levels of PPAR-γ and CEBP were increased upon MYLK knockdown (Fig. 4D and E). Moreover, MYLK siRNA abolished the impact of IGF2BP3 overexpression on adipogenic markers (Fig. 4D and E). Furthermore, we overexpressed MYLK in MSCs (Figure S5A and B), and the results demonstrated that MYLK overexpression exerted the opposite effect, successfully reversing the functional impact of IGF2BP3 knockdown on MSC adipogenesis (Figure S5C-E). These findings provide evidence that IGF2BP3 represses MSC adipogenesis through MYLK.

### IGF2BP3 strengthened the stability of MYLK mRNA

**Fig. 5:**
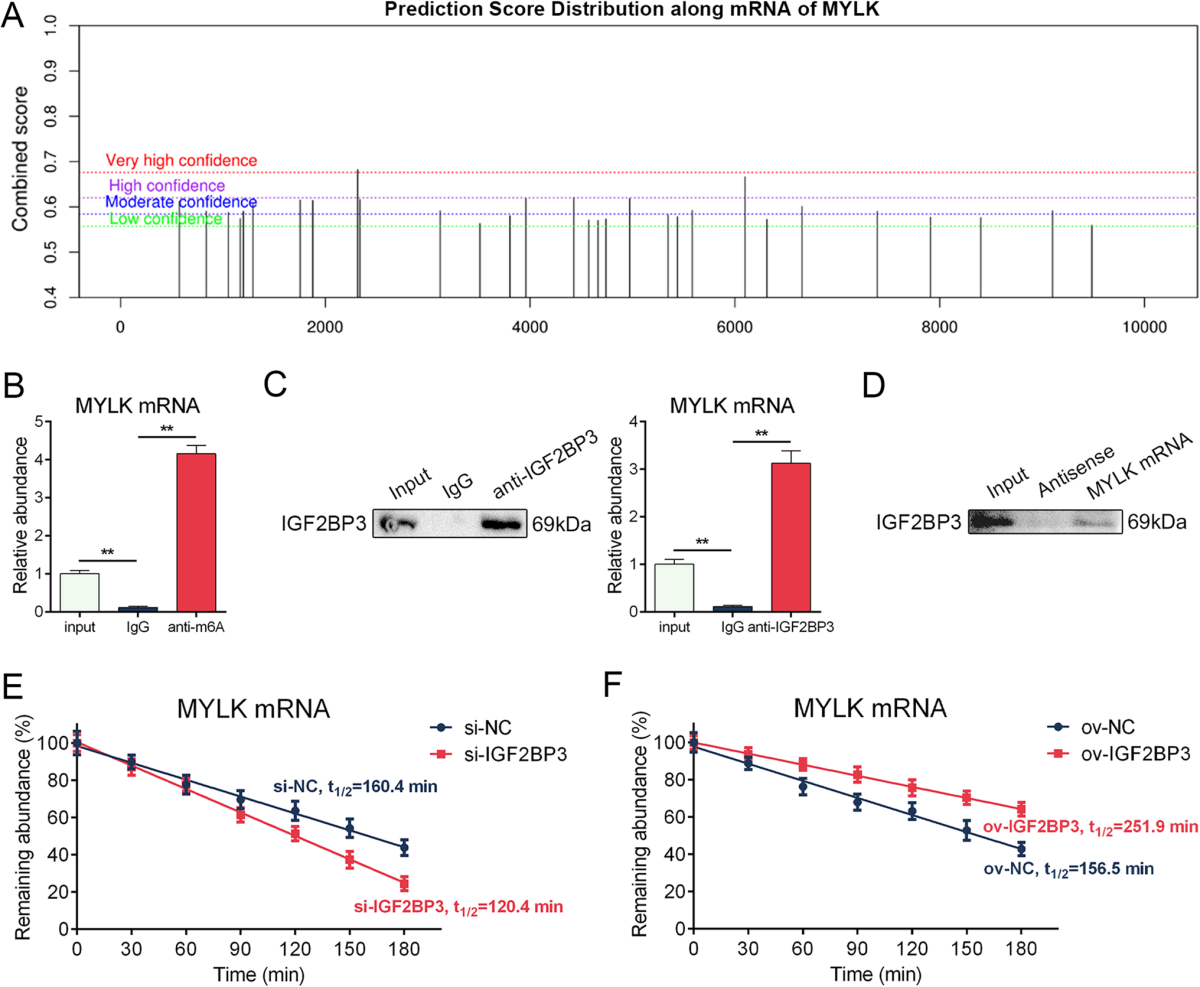
IGF2BP3 strengthened the stability of MYLK mRNA. **A** Prediction of m6A sites on MYLK mRNA using SMARP websites. **B** m6A methylation-RIP-qPCR showed a higher abundance of MYLK mRNA bound to m6A antibody compared to IgG control. **C** RIP-qPCR showed a higher abundance of MYLK mRNA bound to IGF2BP3 compared to IgG control. **D** RNA pulldown revealed the interaction between MYLK mRNA and IGF2BP3 protein. **E** IGF2BP3 knockdown facilitated the degradation of MYLK mRNA. **F** IGF2BP3 overexpression extended the half-life period of MYLK mRNA. *n* = 6 (**B** to **D**), *n* = 12 (**E** and **F**), ** indicates *P* < 0.01

To investigate the mechanism by which IGF2BP3 modulates the level of MYLK, we first predicted the potential m6A sites on MYLK mRNA using SMARP websites [20]. The results revealed the presence of numerous potential m6A sites on MYLK mRNA (Fig. 5A). Furthermore, the RM2Target database [21] showed that several RIP-seq datasets indicated the binding of IGF2BP3 to MYLK mRNA through m6A sites (Figure S6). To validate these findings, we performed m6A methylation-RIP-qPCR, which confirmed the presence of m6A modifications on MYLK mRNA (Fig. 5B). Additionally, RIP-qPCR demonstrated the interaction between IGF2BP3 and MYLK mRNA (Fig. 5C), which was further supported by the RNA pulldown assay (Fig. 5D). These findings suggest that IGF2BP3 regulates the expression of MYLK in an m6A-dependent manner.

It has been reported that IGF2BP3 recognizes m6A modifications on RNA and primarily affects their stability or translation efficiency [11]. Given the regulation of MYLK mRNA level by IGF2BP3, we investigated whether IGF2BP3 influences the degradation rate of MYLK mRNA. We inhibited transcription using actinomycin D and measured the level of MYLK mRNA over time. The results demonstrated that IGF2BP3 knockdown impaired the half-life of MYLK mRNA (Fig. 5E), while IGF2BP3 overexpression prolonged its half-life (Fig. 5F), indicating that IGF2BP3 enhances the stability of MYLK mRNA.

Furthermore, we explored the writer and eraser enzymes involved in the methylation process of MYLK mRNA. The RNA pulldown assay revealed the interaction of the m6A writer METTL3 and the eraser FTO with MYLK mRNA (Figure S7A), which was supported by the RIP assay (Figure S7B). Moreover, we silenced the levels of METTL3 or FTO (Figure S7C and D), and the MeRIP-qPCR assay demonstrated that knockdown of METTL3 reduced while knockdown of FTO enhanced the m6A modification of MYLK mRNA (Figure S7E and F). These results indicate that METTL3 and FTO are the enzymes responsible for the m6A methylation process of MYLK mRNA.

### IGF2BP3 and MYLK repressed MSC adipogenesis via the ERK1/2 pathway

**Fig. 6:**
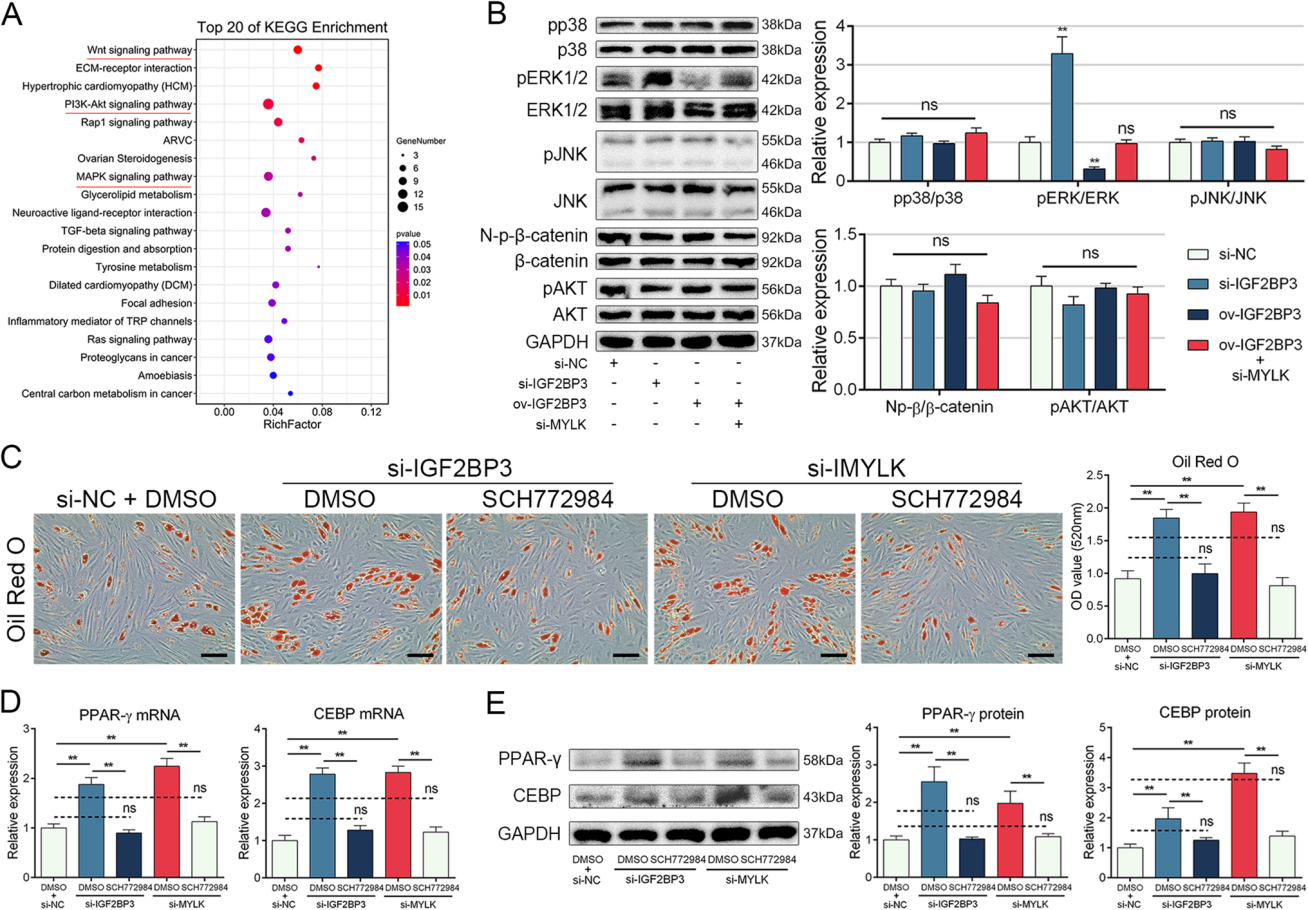
IGF2BP3 and MYLK repressed MSC adipogenesis via ERK1/2 pathway. **A** Top 10 KEGG enrichment results from RNA-seq analysis. **B** Knockdown of IGF2BP3 enhanced phosphorylation of ERK1/2, while IGF2BP3 overexpression weakened phosphorylation of ERK1/2, and this effect was reversed by MYLK knockdown. **C** SCH772984 reversed the effect of IGF2BP3 siRNA and MYLK siRNA on ORO staining. **D** SCH772984 reversed the effect of IGF2BP3 siRNA and MYLK siRNA on the mRNA levels of PPAR-γ and CEBP. **E** SCH772984 reversed the effect of IGF2BP3 siRNA and MYLK siRNA on the protein levels of PPAR-γ and CEBP. *n* = 12, ns indicates not significant, * indicates *P* < 0.05, ** indicates *P* < 0.01, scale bar = 100 nm

To further investigate how MYLK modulates MSC adipogenesis, we examined the enriched KEGG pathways from the RNA-seq analysis mentioned earlier. The top 10 enriched pathways included the Wnt signaling pathway, PI3K-Akt signaling pathway, and MAPK signaling pathway (Fig. 6A), which have been reported to be involved in adipogenesis [22]. Therefore, we assessed the activation of these pathways and observed that knockdown of IGF2BP3 significantly enhanced ERK1/2 phosphorylation, while IGF2BP3 overexpression inhibited it (Fig. 6B). Moreover, MYLK knockdown reversed the effect of IGF2BP3 overexpression (Fig. 6B), and MYLK overexpression rescued the effect of IGF2BP3 knockdown on ERK1/2 phosphorylation (Figure S8).

To further investigate the functional significance of the ERK1/2 pathway, we blocked it by adding SCH772984, a highly selective ERK1/2 inhibitor. The results showed that inhibition of ERK1/2 successfully abolished the effects of IGF2BP3 siRNA and MYLK siRNA on the intensity of ORO staining (Fig. 6C) and the expression of adipogenic markers (Fig. 6D and E). These findings indicate that IGF2BP3 and MYLK repress MSC adipogenesis by inhibiting the ERK1/2 pathway.

### AAVRec2 overexpressing IGF2BP3 lowered body weight and alleviated insulin sensitivity in HFD mice

**Fig. 7:**
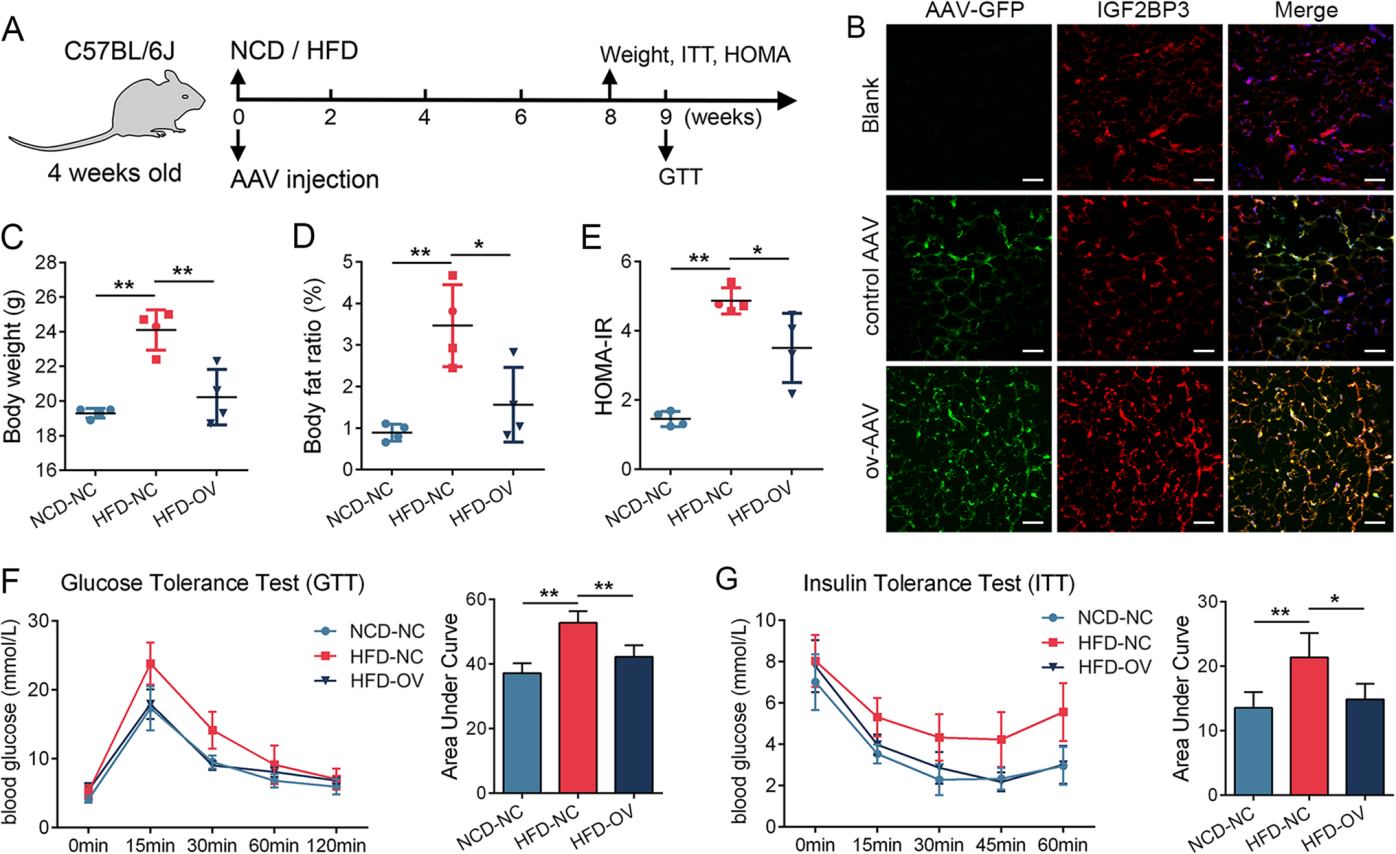
AAVRec2 overexpressing IGF2BP3 lowered the body weight and alleviated insulin sensitivity in HFD mice. **A** The schematic diagram of mice intervention and detection. **B** GFP signal displayed in adipocytes of mice injected with AAV, and stronger IGF2BP3 signal in mice injected with AAVRec2 overexpressing IGF2BP3 (ov-AAV). **C** The body weight was elevated in HFD mice and decreased by AAVRec2 overexpressing IGF2BP3. **D** The body fat ratio was elevated in HFD mice and decreased by AAVRec2 overexpressing IGF2BP3. **E** The HOMA-IR was elevated in HFD mice and decreased by AAVRec2 overexpressing IGF2BP3. **F** The AUC of GTT was elevated in HFD mice and impaired by AAVRec2 overexpressing IGF2BP3. **G.** The AUC of ITT was elevated in HFD mice and impaired by AAVRec2 overexpressing IGF2BP3. *n* = 4, ns indicates not significant, * indicates *P* < 0.05, ** indicates *P* < 0.01, scale bar = 50 nm

Lastly, we assessed the effect of IGF2BP3 on HFD mice. Adeno-associated virus (AAV) is commonly used as a vector for in vivo gene expression and has been applied in clinical treatments [23, 24]. Different AAV serotypes have varying transduction efficiencies in different tissues, and AAVRec2 has been found to exhibit high transduction efficiency in adipose tissue [25]. We constructed HFD mouse models, and IGF2BP3-overexpressing AAVRec2 was intraperitoneally injected (Fig. 7A). The AAVRec2 was transfected, and IGF2BP3 was successfully overexpressed in adipocytes (Fig. 7B). Comparing the HFD mice to the NCD mice, we observed that HFD mice exhibited increased body weight and body fat ratio, which were reduced upon IGF2BP3 overexpression (Fig. 7C and D). Additionally, the insulin resistance index HOMA-IR was elevated in HFD mice but improved with IGF2BP3 overexpression (Fig. 7E). Moreover, IGF2BP3 overexpression also ameliorated glucose tolerance, as measured by the glucose tolerance test (GTT), and enhanced insulin sensitivity, as measured by the insulin tolerance test (ITT) (Fig. 7F and G). These findings indicate that IGF2BP3 helps reduce body weight and improve insulin sensitivity in HFD mice.

## Discussion

In this study, we investigated the involvement of m6A readers in the adipogenic differentiation of MSCs and made a novel discovery that the expression of IGF2BP3 decreases during MSC adipogenesis. We found that IGF2BP3 recognizes and stabilizes MYLK mRNA in an m6A-dependent manner, subsequently inhibiting the phosphorylation of the ERK1/2 pathway and repressing MSC adipogenesis. Furthermore, we observed that overexpression of IGF2BP3 using AAVRec2 resulted in reduced body weight and improved insulin sensitivity in HFD mice. These findings provide valuable insights into MSC adipogenesis-related disorders and potential applications.

M6A is a widely distributed and multifunctional epigenetic modification that has been closely associated with MSC adipogenesis. FTO was the first to be reported to play a crucial role in the regulation of adipogenesis via functioning as a novel regulatory mechanism of RNA processing [26]. In our study, we revealed the involvement of the m6A reader IGF2BP3 in the regulation of MSC adipogenesis. Previous studies have also demonstrated the involvement of several m6A readers in lipogenesis. For instance, IGF2BP2 is essential for early commitment of adipocyte-derived stem cells into preadipocytes, and mice with IGF2BP2 deletion in MSCs exhibit resistance to diet-induced obesity [27]. Similarly, YTHDC2 has been shown to suppress hepatic lipogenesis and TG homeostasis, making it a potential target for treating liver steatosis and insulin resistance [28]. In contrast to these previous studies, our research comprehensively explored the expression of m6A readers during MSC adipogenesis and revealed, for the first time, the gradual decrease in IGF2BP3 levels upon induction of adipogenic differentiation. This suggests that IGF2BP3 may act as a protective factor preventing MSCs from undergoing adipogenesis in a normal environment but is displaced upon adipogenic induction.

Additionally, we discovered that IGF2BP3 represses MSC adipogenesis by enhancing the expression of MYLK. Readers recognize m6A modifications and exert various effects on RNA [10]. IGF2BPs have been shown to slow degradation rates, enhance translation efficiency, and regulate the cellular localization of transcripts [29]. Consistently, our study demonstrated that IGF2BP3 interacts with MYLK mRNA, promoting its stability. Previously, Zhao et al. reported that the m6A methyltransferase METTL3 alleviates degradation of MYLK mRNA through m6A modification, highlighting the importance of m6A modification in MYLK mRNA stability [30]. However, Zhao's study did not investigate the involvement of relevant m6A readers, and our study is the first to identify IGF2BP3 as an m6A reader of MYLK mRNA.

MYLK, a phosphorylation kinase primarily involved in muscle contractility [31], has recently been linked to the development and treatment of various inflammatory injuries and malignancies [32–34]. For instance, MYLK has been found to promote ovarian cancer cell motility and metastasis [32]. However, its role in MSCs remains poorly understood. Only Lin's study has reported that MYLK is activated by IL-1β and drives MSC migration to injury sites [16]. In our investigation, we observed a decrease in MYLK levels during adipogenic induction and discovered its inhibitory effect on MSC adipogenesis. These findings suggest that MYLK plays a crucial role in regulating MSC function and may participate in other biological processes, warranting further research.

Our results indicated that MYLK suppresses MSC adipogenesis by inhibiting the activation of the ERK pathway, a well-known signaling pathway involved in adipogenic differentiation [35]. Previous studies have reported an interaction of MYLK and ERK pathways, but the results have been inconsistent. Kim et al. reported that decreased expression of MYLK increased the level of phosphorylated ERK1/2 in breast epithelial cells [36], which aligns with our findings. In contrast, Bessard et al. demonstrated that MYLK inhibition did not impede ERK1/2 phosphorylation in pulmonary arterial endothelial cells [37], while Anis et al. showed that MYLK contributed to endothelial cell hyperproliferation by activating the ERK pathway [38]. It is plausible that the effect of MYLK on the ERK pathway may vary in different cell lines or cellular states.

MSCs, as the primary source of adipocytes, play a crucial role in adipose tissue, which accounts for a significant proportion of total body mass in lean adults. Adipose tissue serves as a vital energy storage and metabolic organ, as well as exhibiting endocrine functions [35]. Abnormal adipocyte formation is implicated in various pathological processes, including obesity, type 2 diabetes, fatty liver, and cardiovascular diseases [39]. Therefore, understanding the regulatory network of MSC adipogenesis in both physiological and pathological states is of great significance. In this study, we first elucidated the role of the IGF2BP3-MYLK-ERK1/2 axis in physiological MSC adipogenesis, shedding light on the underlying mechanism and providing potential regulatory targets for controlling MSC adipogenesis. However, the dysregulation of this axis in pathological conditions and its involvement in related disorders remain unclear and warrant further investigation.

Furthermore, we evaluated the impact of IGF2BP3 on HFD mice by employing AAVRec2 as a delivery system. Our results demonstrated that IGF2BP3 expression helped reduce body fat ratio, body weight, and alleviate insulin resistance. Similar findings have been reported for GPNMB and LGR1, which were shown to mediate obesity and insulin resistance by promoting lipogenesis [40, 41]. HFD is known to induce excessive fat accumulation and increase adiposity, contributing to dysfunctional obese adipose tissue and insulin resistance [42]. Adipose tissue secretes various bioactive molecules, including peptide hormones, cytokines, and activated lipids, which can influence energy metabolism in other tissues and contribute to insulin resistance [43]. Combining our in vitro experiments and in vivo adipogenesis assay on nude mice, we propose that IGF2BP3 reduces body weight and insulin resistance by inhibiting adipocyte production in HFD mice, at least partially. However, it remains unclear whether IGF2BP3 affects other functions of adipocytes, such as lipolysis or adipokine secretion, which necessitates further investigation. Collectively, these findings suggest that targeting adipogenesis could be a potential strategy for managing weight gain and insulin resistance. Additionally, our data demonstrated the successful expression of IGF2BP3 in adipocytes of mice through intraperitoneal injection of AAVRec2. This aligns with the findings of Huang's research, which highlighted the high transduction efficiency of AAVRec2 in adipose tissue [28]. Previously, the application of AAV in adipose tissue was limited by low transduction efficiency, and the emergence of AAVRec2 as a highly efficient delivery system represents a significant advancement in gene therapy targeting adipocytes.

MSCs possess several characteristics, including easy accessibility, self-renewal, multilineage differentiation potential, and low immunogenicity, which render them highly valuable and promising in the field of biomedical engineering [44]. Among the various properties of MSCs, adipogenesis has garnered significant attention and found widespread applications, such as in the repair of soft tissue defects and breast augmentation [7, 45]. Studies conducted by Seung-Woo and colleagues have demonstrated the effective generation of adipose tissue from MSCs, suggesting their potential as a safe and efficient therapeutic approach for soft tissue regeneration [6]. Furthermore, several clinical trials have reported the successful use of MSC-derived adipocytes for soft tissue augmentation, with relatively long-lasting volume maintenance [46, 47]. In our study, we have identified interfering with IGF2BP3 as an effective strategy to enhance MSC adipogenesis. Additionally, our in vivo experiments on mice have shown that targeting IGF2BP3 can modulate fat formation. These findings highlight IGF2BP3 as a potential regulatory target for applications related to MSC adipogenesis. However, further investigation is necessary to explore the full potential and specific mechanisms underlying the role of IGF2BP3 in MSC adipogenesis-related applications.

In conclusion, we revealed a new regulatory axis, IGF2BP3-MYLK-ERK1/2, in the adipogenic differentiation of MSCs and provided new insights for the investigation of adipogenesis-related disorders and applications.

### Supplementary Information

Below is the link to the electronic supplementary material.

Supplementary file1 (TIF 5376 KB)

Supplementary file2 (TIF 6223 KB)

Supplementary file3 (TIF 5549 KB)

Supplementary file4 (TIF 4663 KB)

Supplementary file5 (TIF 5389 KB)

Supplementary file6 (TIF 3607 KB)

Supplementary file7 (TIF 6376 KB)

Supplementary file8 (TIF 1368 KB)

Supplementary file9 (DOCX 27 KB)

## Data Availability

The data in this study are available from the corresponding authors on reasonable request.
