# Supplementary figures and images for: IGF2BP3-mediated enhanced stability of MYLK represses MSC adipogenesis and alleviates obesity and insulin resistance in HFD mice
Source: Cell Mol Life Sci. 2024 Jan 10;81(1):17. doi: 10.1007/s00018-023-05076-0 (PMC10776757; doi:10.1007/s00018-023-05076-0)

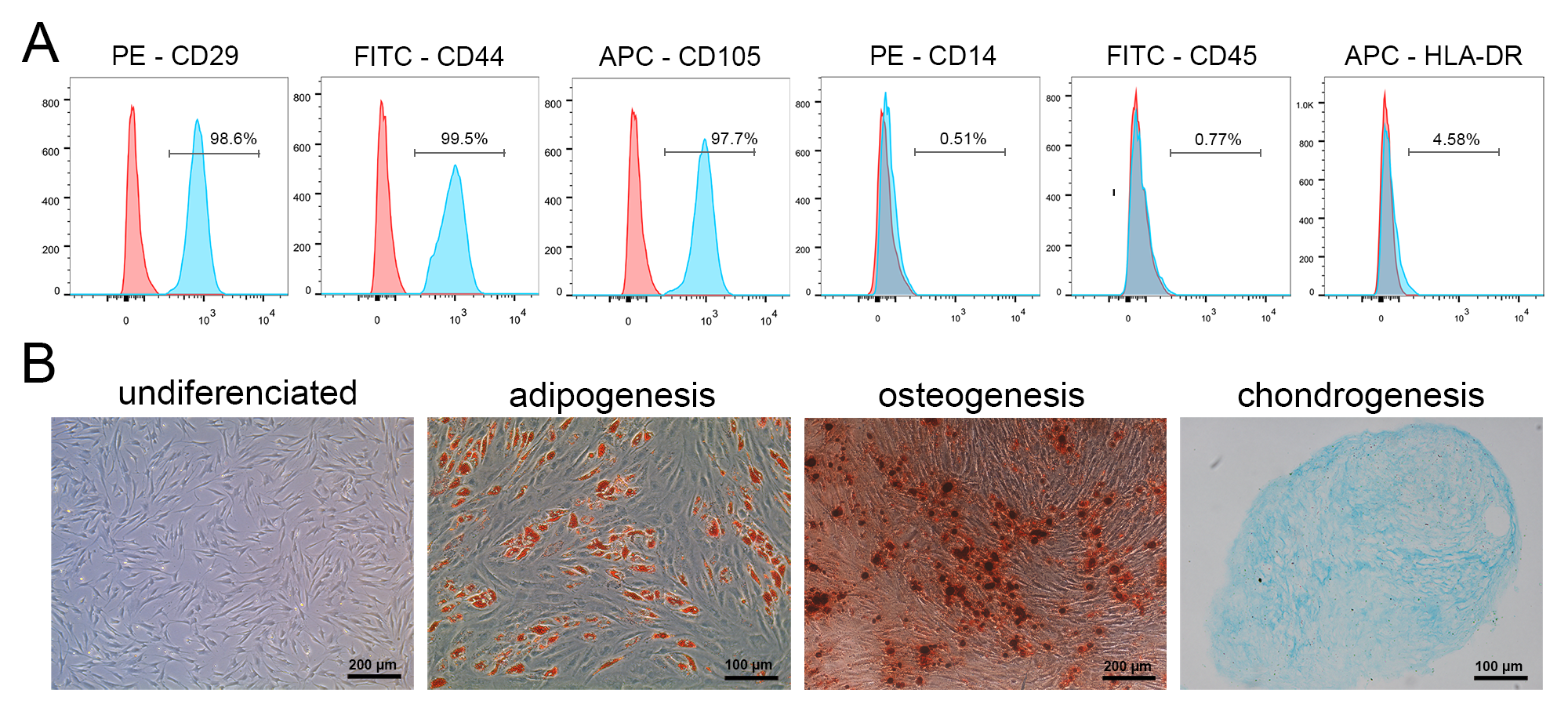

Supplement: Supplementary file 1 — Supplementary file1 (TIF 5376 KB) [file 18_2023_5076_MOESM1_ESM.tif]

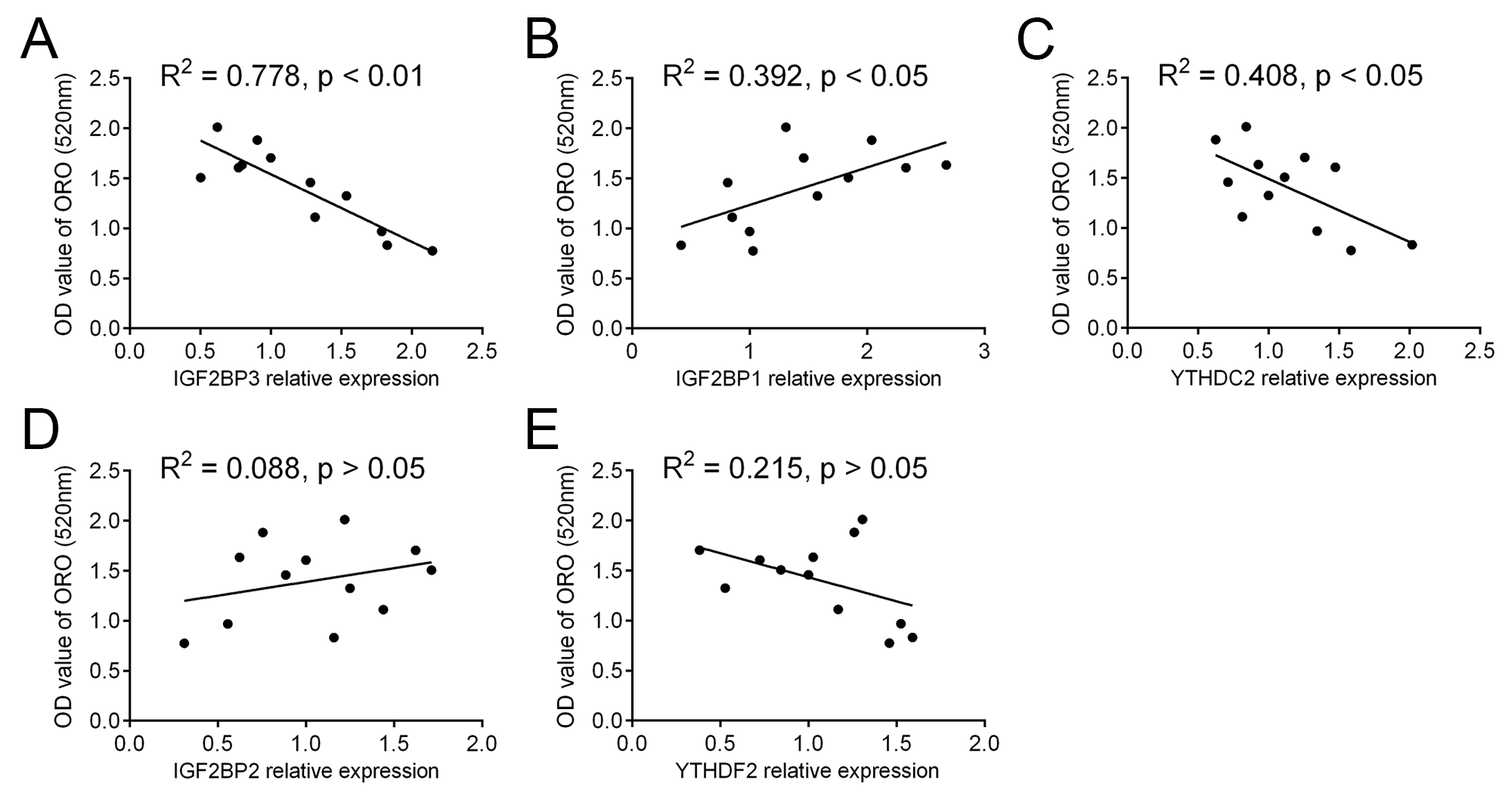

Supplement: Supplementary file 2 — Supplementary file2 (TIF 6223 KB) [file 18_2023_5076_MOESM2_ESM.tif]

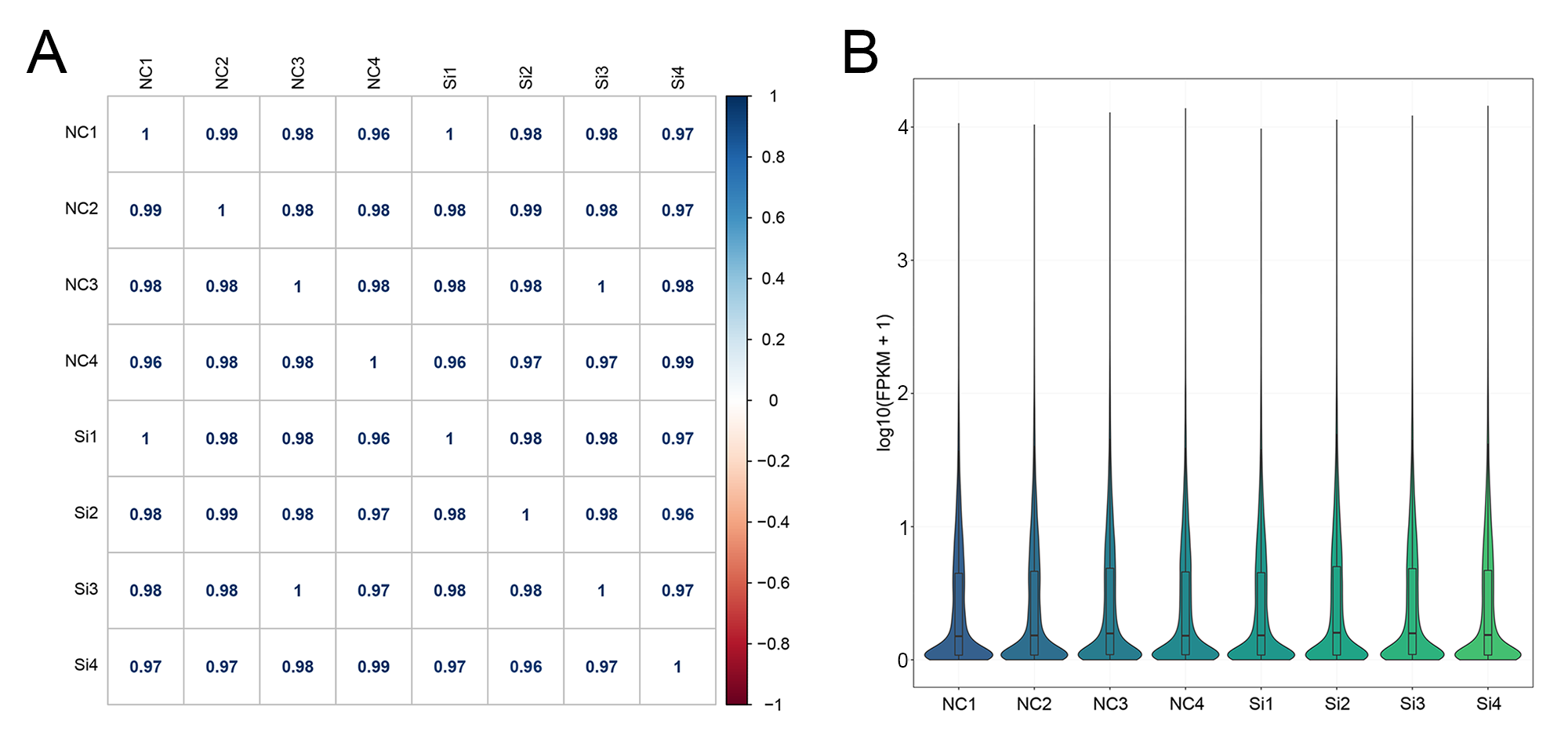

Supplement: Supplementary file 3 — Supplementary file3 (TIF 5549 KB) [file 18_2023_5076_MOESM3_ESM.tif]

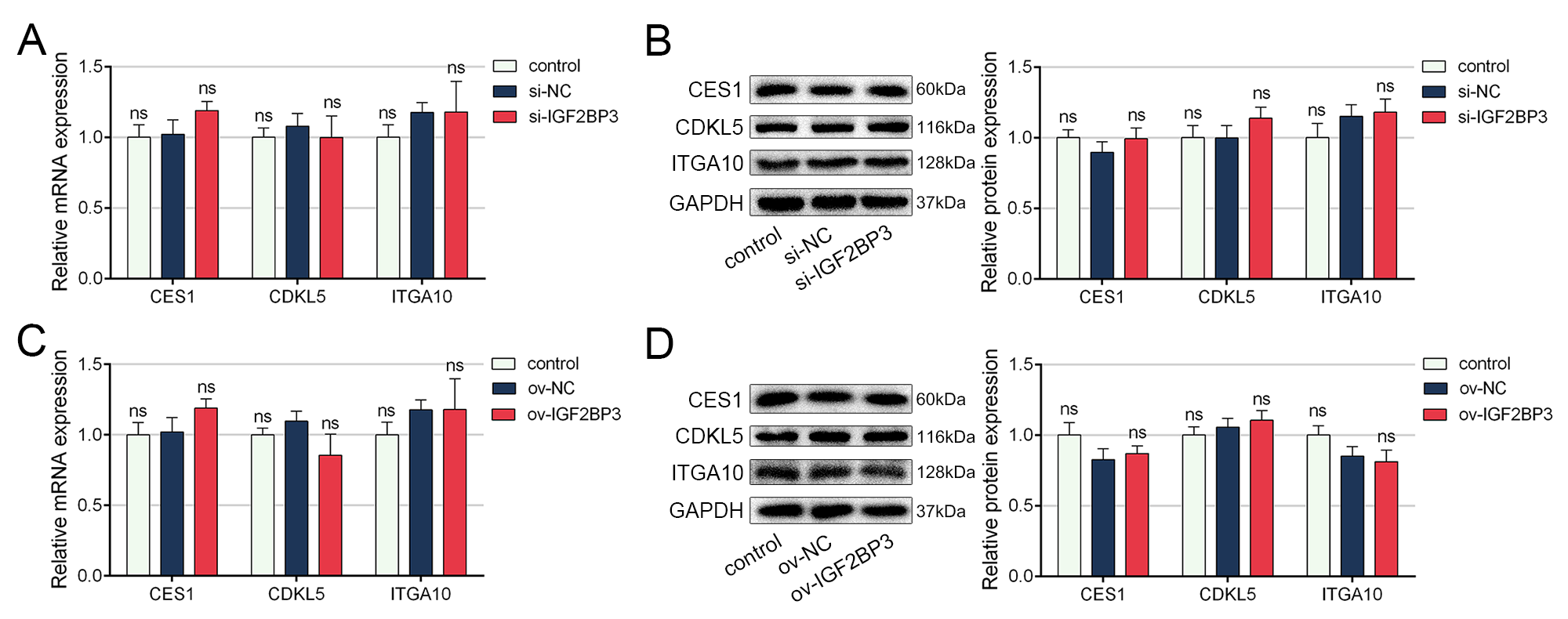

Supplement: Supplementary file 4 — Supplementary file4 (TIF 4663 KB) [file 18_2023_5076_MOESM4_ESM.tif]

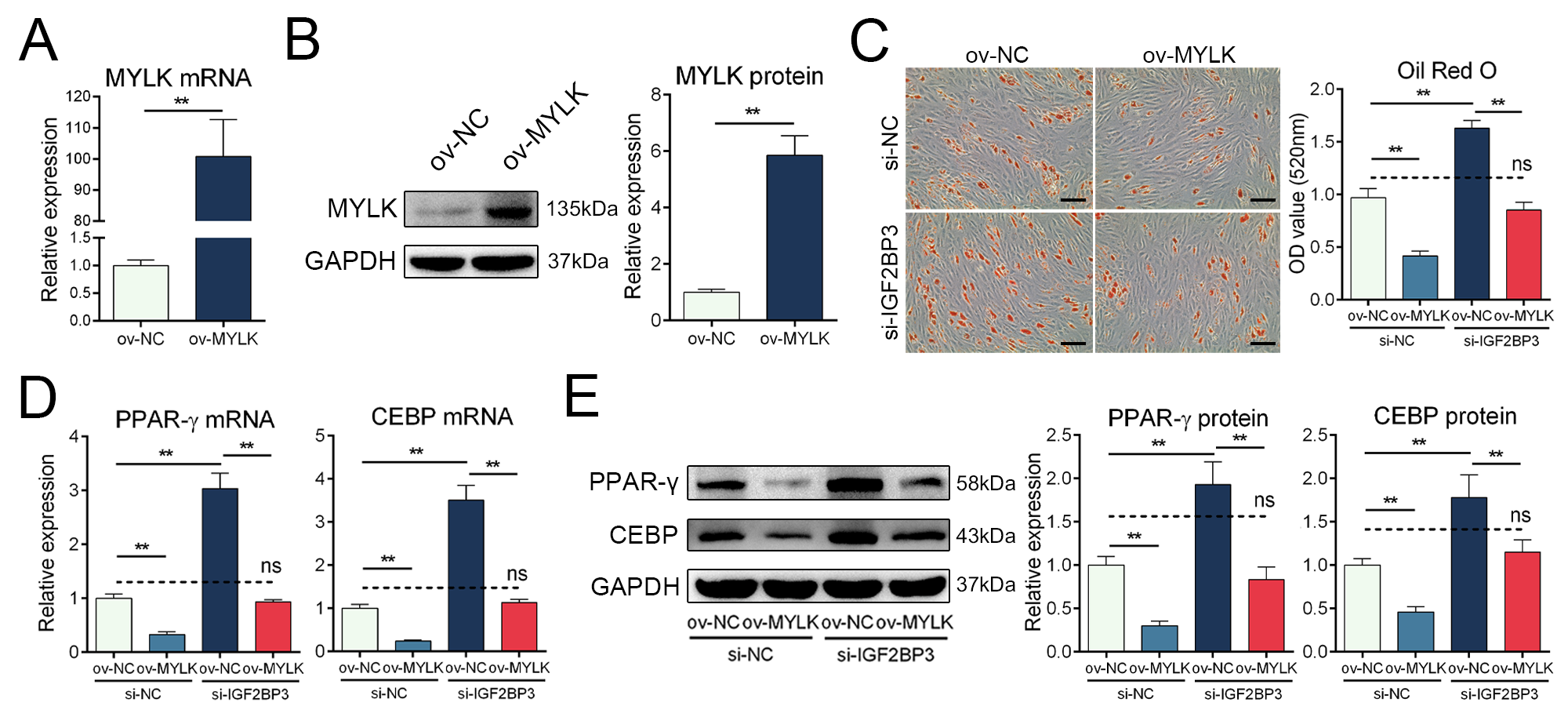

Supplement: Supplementary file 5 — Supplementary file5 (TIF 5389 KB) [file 18_2023_5076_MOESM5_ESM.tif]

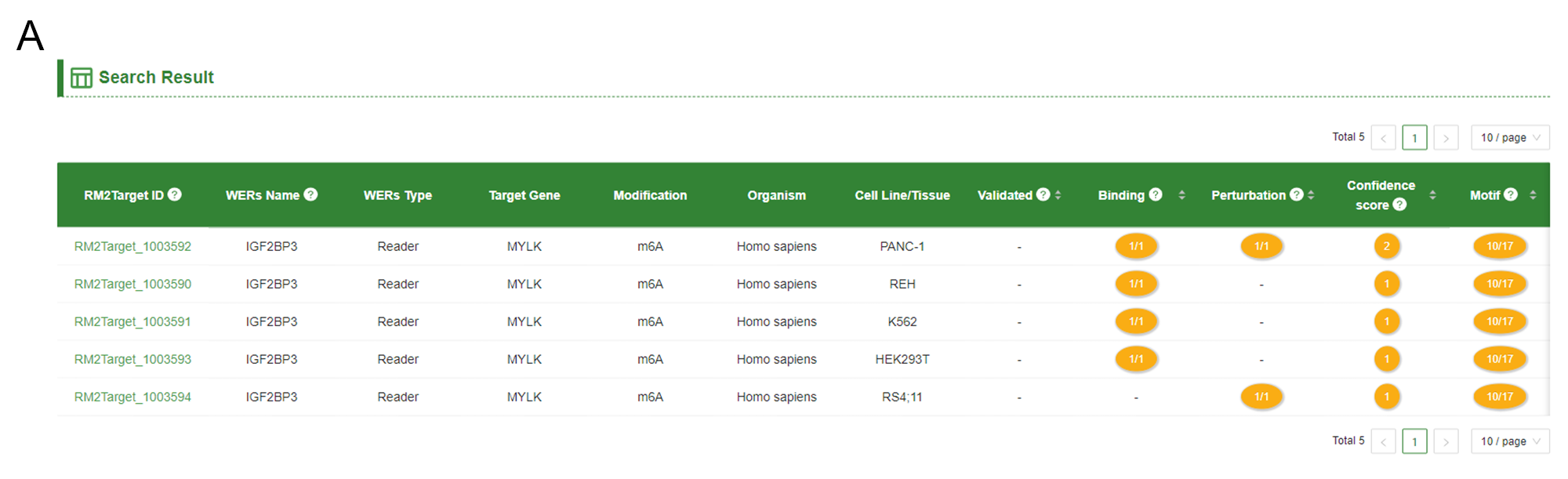

Supplement: Supplementary file 6 — Supplementary file6 (TIF 3607 KB) [file 18_2023_5076_MOESM6_ESM.tif]

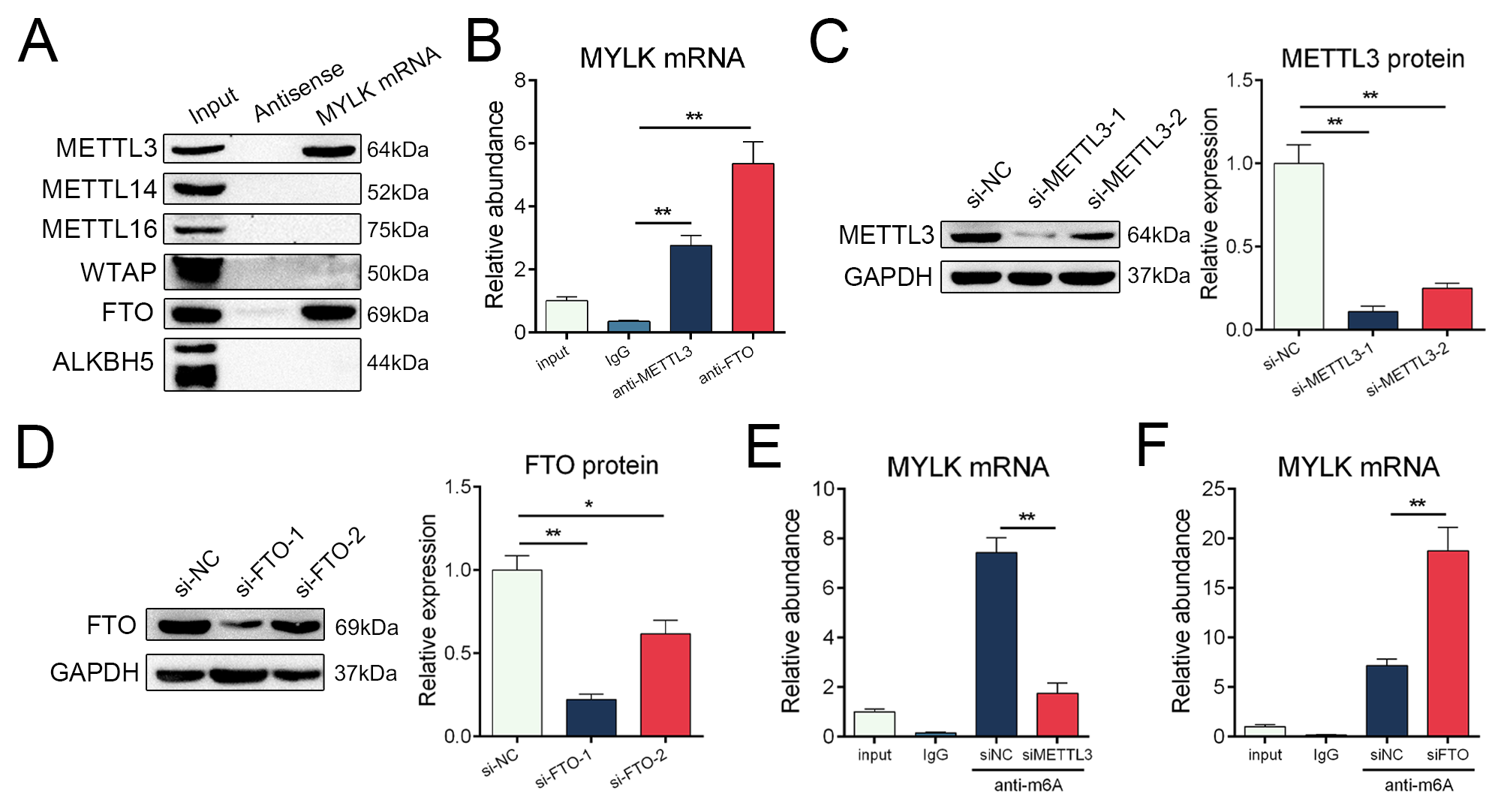

Supplement: Supplementary file 7 — Supplementary file7 (TIF 6376 KB) [file 18_2023_5076_MOESM7_ESM.tif]

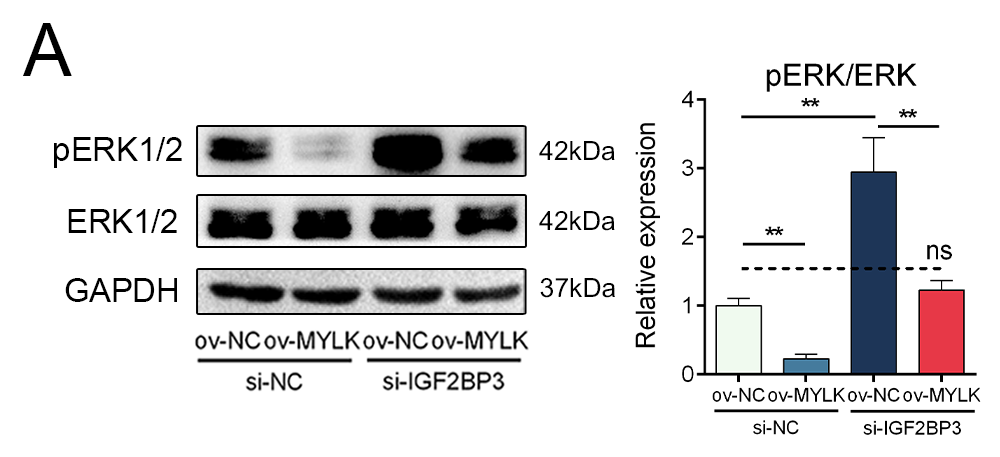

Supplement: Supplementary file 8 — Supplementary file8 (TIF 1368 KB) [file 18_2023_5076_MOESM8_ESM.tif]
