# Supplementary material for: IGF2BP3-mediated enhanced stability of MYLK represses MSC adipogenesis and alleviates obesity and insulin resistance in HFD mice
Source: Cell Mol Life Sci. 2024 Jan 10;81(1):17. doi: 10.1007/s00018-023-05076-0 (PMC10776757; doi:10.1007/s00018-023-05076-0)
Supplement: Supplementary file 9 — Supplementary file9 (DOCX 27 KB) [file 18_2023_5076_MOESM9_ESM.docx]

**Supplementary Tables**

**Table S1. Primers used for qRT-PCR**

| **Gene** | **Forward primer (5' - 3')** | **Reverse primer (5' - 3')** |
| --- | --- | --- |
| GAPDH | AAGGTGAAGGTCGGAGTCAA | AATGAAGGGGTCATTGATGG |
| YTHDC1 | CTTCTGATGAGCAAGGGAACAA | GGCCTCACTTCGAGTGTCATAA |
| YTHDC2 | CAAAACATGCTGTTAGGAGCCT | CCACTTGTCTTGCTCATTTCCC |
| YTHDF1 | ACCTGTCCAGCTATTACCCG | TGGTGAGGTATGGAATCGGAG |
| YTHDF2 | CCTTAGGTGGAGCCATGATTG | TCTGTGCTACCCAACTTCAGT |
| YTHDF3 | GGTGTATTTAGTCAACCTGGGG | AAGAGAACTAGGTGGATAGCCAT |
| IGF2BP1 | GGCCATCGAGAATTGTTGCAG | CCAGGGATCAGGTGAGACTG |
| IGF2BP2 | AGCTAAGCGGGCATCAGTTTG | CCGCAGCGGGAAATCAATCT |
| IGF2BP3 | TATATCGGAAACCTCAGCGAGA | GGACCGAGTGCTCAACTTCT |
| PPAR-γ | ACCAAAGTGCAATCAAAGTGGA | ATGAGGGAGTTGGAAGGCTCT |
| CEBP | TATAGGCTGGGCTTCCCCTT | AGCTTTCTGGTGTGACTCGG |
| SAA1 | GAGATTCTTTGGCCATGGTGC | CCAGCAGGTCGGAAGTGATT |
| MYLK | CCCGAGGTTGTCTGGTTCAAA | GCAGGTGTACTTGGCATCGT |
| CES1 | ACCCCTGAGGTTTACTCCACC | TGCACATAGGAGGGTACGAGG |
| CDKL5 | GCAGGAAAACATTGTGGAGTTG | GCACCAGTGAATAGCCTTGATTA |
| ITGA10 | AACATCACCCACGCCTATTCC | GTTGGTAGTCACCTAAGTGGC |

**Table S2. siRNAs used for RNA interference**

| **Target Gene** | **siRNA sequence (5' - 3')** |
| --- | --- |
| si-NC | UUCUCCGAACGUGUCACGUTT |
| si-IGF2BP3-1 | CGGUGAAUGAACUUCAGAATT |
| si-IGF2BP3-2 | GCUGCUGAGAAGUCGAUUATT |
| si-MYLK-1 | GGAAGUUCUUGUUUAUAUATT |
| si-MYLK-2 | GGAAGUUCUUCAAGGCAUATT |
| si-METTL3-1 | GAGUGAUAUUUGUACAAUATT |
| si-METTL3-2 | CAGUGGAUCUGUUGUGAUATT |
| si-FTO-1 | GCUUGGUGUUAAAGAUUAATT |
| si-FTO-2 | GAUUCUAGUUACAGACUUATT |

**Table S3. Information of used primary antibodies**

| **Target** | **Company** | **Catalog #** | **Dilution** |
| --- | --- | --- | --- |
| GAPDH | Abcam | ab8245 | 1:5000 for WB |
| YTHDC1 | Abcam | ab259990 | 1:1000 for WB |
| YTHDC2 | Abcam | ab220160 | 1:1000 for WB |
| YTHDF1 | Abcam | ab252346 | 1:1000 for WB |
| YTHDF2 | Abcam | ab220163 | 1:1000 for WB |
| YTHDF3 | Abcam | ab220161 | 1:1000 for WB |
| IGF2BP1 | Abcam | ab184305 | 1:1000 for WB |
| IGF2BP2 | Abcam | ab128175 | 1:1000 for WB |
| IGF2BP3 | Abcam | ab177477 | 1:1000 for WB  1:100 for RIP |
|  |  |  | 1:100 for IF |
| METTL3 | Abcam | ab195352 | 1:1000 for WB |
|  |  |  | 1:50 for RIP |
| FTO | Proteintech | 27226-1-AP | 1:1000 for WB |
|  |  |  | 1:50 for RIP |
| SAA1 | Proteintech | 13192-1-AP | 1:1000 for WB |
| MYLK | Proteintech | 21642-1-AP | 1:1000 for WB |
| CES1 | Proteintech | 16912-1-AP | 1:1000 for WB |
| CDKL5 | Proteintech | 12973-1-AP | 1:1000 for WB |
| ITGA10 | Signalway Antibody | #44824 | 1:1000 for WB |
| PPAR-γ | Cell Signaling Technology | #2443 | 1:1000 for WB |
| C/EBP-α | Cell Signaling Technology | #2295 | 1:1000 for WB |
| p38 | Cell Signaling Technology | #8690 | 1:1000 for WB |
| pp38 | Cell Signaling Technology | #4511 | 1:1000 for WB |
| p38 | Cell Signaling Technology | #8690 | 1:1000 for WB |
| pp38 | Cell Signaling Technology | #4511 | 1:1000 for WB |
| ERK1/2 | Cell Signaling Technology | #4695 | 1:1000 for WB |
| pERK1/2 | Cell Signaling Technology | #4370 | 1:1000 for WB |
| JNK | Cell Signaling Technology | #9252 | 1:1000 for WB |
| pJNK | Cell Signaling Technology | #9255 | 1:1000 for WB |
| β-catenin | Cell Signaling Technology | #9582 | 1:1000 for WB |
| N-p-β-catenin | Cell Signaling Technology | #4270 | 1:1000 for WB |
| AKT | Cell Signaling Technology | #2920 | 1:1000 for WB |
| pAKT | Cell Signaling Technology | #4060 | 1:1000 for WB |
| Perilipin-1 | Abcam | ab3526 | 1:100 for IHC |
| m6A methylation | Synaptic Systems | 202003 | 1:100 for RIP |

Abbreviations: WB, Western Blot; RIP, RNA Immunoprecipitation; IHC, Immunohistochemistry, IF, Immunofluorescence.

**Supplementary Figure Legends**

**Figure S1.** Characteristics of MSCs. **A.** Flow cytometry analysis demonstrated that the majority of cells were positive for CD29, CD44, and CD105, while negative for CD14, CD45, and HLA-DR. **B.** Alizarin Red S, Oil Red O, and toluidine blue staining showed the multi-lineage potential of the cells, with the ability to differentiate into adipocytes, osteoblasts, and chondroblasts, respectively. n = 12.

**Figure S2.** Correlation analysis of the readers levels and the strength of ORO staining. **A.** The correlation analysis showed that the R-squared value between the level of IGF2BP3 and the strength of ORO staining was 0.778. **B.** The correlation analysis showed that the R-squared value between the level of IGF2BP1 and the strength of ORO staining was 0.392. **C.** The correlation analysis showed that the R-squared value between the level of YTHDC2 and the strength of ORO staining was 0.408. **D.** The correlation analysis showed that the R-squared value between the level of IGF2BP2 and the strength of ORO staining was 0.088. **E.** The correlation analysis showed that the R-squared value between the level of YTHDF2 and the strength of ORO staining was 0.215. n = 12.

**Figure S3.** Expression distribution of the RNA-seq samples. **A.** The Corplot diagram showed the correlation between all samples. **B.** The violin diagram depicts the gene expression levels, highlighting the distribution and variation across the samples.

**Figure S4.** IGF2BP3 did not affect the levels of CES1, CDKL5 and ITGA10. **A - B.** Both the mRNA and protein levels of CES1, CDKL5 and ITGA10 were not affected by IGF2BP3 knockdown. **C - D.** Both the mRNA and protein levels of CES1, CDKL5 and ITGA10 were not affected by IGF2BP3 overexpression. n = 6, ns indicates not significant.

**Figure S5.** MYLK overexpression inhibited MSC adipogenesis and reversed the effect of IGF2BP3 knockdown. **A.** The overexpression efficiency of MYLK lentivirus on mRNA level. **B.** The overexpression efficiency of MYLK lentivirus on protein level. **C.** MYLK overexpression inhibited the intensity of ORO staining and reversed the effect of IGF2BP3 knockdown. **D.** mRNA levels of PPAR-γ and CEBP are inhibited by MYLK overexpression and reversed by IGF2BP3 knockdown. **E.** Protein levels of PPAR-γ and CEBP are inhibited by MYLK overexpression and reversed by IGF2BP3 knockdown. n = 12, ns indicates not significant, * indicates P < 0.05, ** indicates P < 0.01.

**Figure S6.** Search results on RM2Target database. **A.** Multiple datasets of RIP-seq analysis showed the binding of IGF2BP3 to MYLK mRNA through the m6A sites.

**Figure S7.** METTL3 and FTO regulated the m6A methylation level of MYLK mRNA. **A.** METTL3 protein and FTO protein were integrated with MYLK mRNA. **B.** MYLK mRNA was precipitated by METTL3 antibody and FTO antibody. **C.** Interference efficiency of METTL3 siRNAs on protein level. **D.** Interference efficiency of FTO siRNAs on protein level. **E.** Knockdown of METTL3 reduced the m6A modification level of MYLK mRNA. **F.** Knockdown of FTO enhanced the m6A modification level of MYLK mRNA. n = 6, ns indicates not significant, * indicates P < 0.05, ** indicates P < 0.01.

**Figure S8.** MYLK overexpression inhibited the activation of ERK1/2 pathway. **A.** MYLK overexpression inhibited the activation of ERK1/2 pathway and reversed the effect of IGF2BP3 knockdown. n = 12, ns indicates not significant, ** indicates P < 0.01.
